# Lower Extremity Peripheral Arterial Disease Is an Independent Predictor of Coronary Heart Disease and Stroke Risks in Patients with Type 2 Diabetes Mellitus in China

**DOI:** 10.1155/2017/9620513

**Published:** 2017-05-08

**Authors:** Xiao-Hong Pang, Jue Han, Wan-Lan Ye, Xue Sun, Yue Ding, Wen-Juan Huang, Yi-Ming Zhao, Han-Yu Lou, Li-Zhen Shan, Ying-Xiu Kang, Xiao-Xiao Song, Song-Zhao Zhang, Wei Gu, Peng-Fei Shan

**Affiliations:** ^1^Department of Endocrinology and Metabolism, The Second Affiliated Hospital Zhejiang University College of Medicine, 88 Jiefang Rd, Hangzhou, Zhejiang 310009, China; ^2^Department of Clinical Laboratory, The Second Affiliated Hospital Zhejiang University College of Medicine, 88 Jiefang Rd, Hangzhou, Zhejiang 310009, China

## Abstract

We aimed to determine the relationship between lower extremity peripheral arterial disease (PAD), 10-year coronary heart disease (CHD), and stroke risks in patients with type 2 diabetes (T2DM) using the UKPDS risk engine. We enrolled 1178 hospitalized T2DM patients. The patients were divided into a lower extremity PAD group (ankle-brachial index ≤ 0.9 or >1.4; 88 patients, 7.5%) and a non-PAD group (ankle-brachial index > 0.9 and ≤1.4; 1090 patients, 92.5%). Age; duration of diabetes; systolic blood pressure; the hypertension rate; the use of hypertension drugs, ACEI /ARB, statins; CHD risk; fatal CHD risk; stroke risk; and fatal stroke risk were significantly higher in the PAD group than in the non-PAD group (*P* < 0.05 for all). Logistic stepwise regression analysis indicated that ABI was an independent predictor of 10-year CHD and stroke risks in T2DM patients. Compared with those in the T2DM non-PAD group, the odds ratios (ORs) for CHD and stroke risk were 3.6 (95% confidence interval (CI), 2.2–6.0; *P* < 0.001) and 6.9 (95% CI, 4.0–11.8; *P* < 0.001) in those with lower extremity PAD, respectively. In conclusion, lower extremity PAD increased coronary heart disease and stroke risks in T2DM.

## 1. Introduction

Diabetes patients with peripheral arterial disease (PAD) are at an increased risk for cardiovascular disease [1, 2]. Lower extremity peripheral arterial disease (PAD) is a common type of PAD in patients with type 2 diabetes mellitus (T2DM). In diabetic patients, PAD can be noninvasively and objectively diagnosed by using the ankle-brachial index (ABI); this index can also indicate arterial atherosclerosis at other sites [3, 4]. A low ABI is related to many known cardiovascular risk factors, including hypertension, diabetes, smoking, dyslipidemia, obesity, and increased serum levels of C-reactive protein [5–7]. A few population-based cohort studies have confirmed that a decrease in the ABI is highly correlated with an increase in the prevalence rate of coronary artery disease and cerebrovascular disease [8–11].

Currently, a number of methods are available for predicting the 10-year risk of cardiovascular disease in individual subjects, such as the Framingham Risk Score (FRS), the 2013 American College of Cardiology (ACC)/American Heart Association (AHA) risk assessment, and the United Kingdom Prospective Diabetes Study (UKPDS) risk engine. The FRS was derived from the Framingham Heart Study to assess the cardiovascular risk based on age, low-density lipoprotein cholesterol (LDL-c), high-density lipoprotein cholesterol (HDL-c), smoking, hypertension, and other factors [12]. Since the FRS was generated using data from the general population, its usefulness in predicting cardiovascular risk in diabetic patients is somewhat limited [13]. The 2013 ACC/AHA risk assessment applies to non-Hispanic American men aged 40–79 years [14]. The UKPDS risk engine, on the other hand, is the commonly used method for the prediction of cardiovascular and cerebrovascular disease risk in T2DM patients. This diabetes-specific risk assessment tool is based on the absolute risk of cardiovascular and cerebrovascular diseases in 5102 patients with newly diagnosed T2DM who were followed up for an average of 10.4 years [15]. Unlike the FRS, the UKPDS risk engine takes into consideration the duration of diabetes and the level of glycosylated hemoglobin (HbA1c).

Some reports have indicated that the ABI abnormality was linked to cardiovascular events, cerebrovascular events, and risk factors in patients with diabetes or metabolic syndrome [1, 9, 16, 17]. A study from Hong Kong found that in diabetic patients with a slightly decreased ABI (0.91–0.99), the ABI was associated with increased microvascular and macrovascular complications [18]. Mainland China has the largest population of diabetes patients in the world; however, few studies have investigated the relationship of the ABI with the 10-year coronary heart disease (CHD) and stroke risks in T2DM patients in Mainland China. In this study, we aimed to characterize the above relationship in T2DM patients in China by using the UKPDS risk engine.

## 2. Materials and Methods

### 2.1. Subjects

This study involved T2DM patients who were admitted to the Department of Endocrinology of the Second Affiliated Hospital Zhejiang University Medical College between April 2008 and April 2013. All participants had been diagnosed with diabetes according to the 1999 World Health Organization diagnostic criteria for the diagnosis and classification of diabetes. In our study, we involved only type 2 diabetes mellitus patients. Those with gestational diabetes, other types of diabetes mellitus, type 1 diabetes mellitus, GAD antibody positivity were excluded. Further, patients with CHD and stroke were additionally excluded, leaving a total of 1178 cases that were included in the statistical analysis. This study was approved by the Ethics Committee of the Second Affiliated Hospital Zhejiang University School of Medicine, and all subjects gave informed consent for participation.

### 2.2. Clinical Indices

A detailed medical history was obtained from each patient, including the patient's age, age at diagnosis of diabetes, smoking history, hypertension, and antihypertensive therapy. Each subject also underwent a detailed physical examination, including height, weight, blood pressure, and body mass index (BMI) measurements. Prior to the blood pressure measurements, the patients were asked to sit for 5 min. Subsequently, two consecutive blood pressure measurements were taken with an electronic blood pressure meter (Kenz BPM SP-1, Japan), and the mean of the two values was used.

### 2.3. Biochemical Indices

Venous blood was collected in the morning (6:00–9:00 AM) after the patient had fasted for 8–12 hours. The fasting blood glucose, total cholesterol (TC), triglyceride (TG), LDL-c, and HDL-c levels were measured by an Olympus AU4500 automatic chemistry analyzer (Olympus Corporation, Tokyo, Japan). The level of HbA1c was determined by a TOSOH HLC-723G8 automatic glycohemoglobin analyzer (Tosoh Corporation, Yamaguchi 746-0042, Japan).

### 2.4. ABI Measurement

The ABI was measured by a technician who was blinded to the patient history and biochemical indices. The ABI was determined using Doppler ultrasound and a portable optical volume detector (Vista AVS, Summit Doppler, USA). The patients were asked to take off their shoes and lie in a supine position for 5 min. The upper arm and ankle systolic pressures were measured by slowly moving the ultrasonic probe along the arterial contorts until the strongest information was gotten. The ABI was calculated as the ratio of the ankle systolic blood pressure to the brachial arterial systolic pressure. Blood pressure was measured in both lower extremities and used to calculate the ABI. The lower of the two ABI values thus obtained was used in the subsequent analyses, unless one of the ABI values was greater than 1.4. The patients were divided into two groups based on the ABI value as follows: patients with an ABI ≤ 0.9 or ABI > 1.4 were assigned to the PAD group and those with an ABI > 0.9 and ≤1.4 formed the non-PAD group [3].

### 2.5. UKPDS Risk Engine

The risks of CHD, fatal CHD, stroke, and fatal stroke were calculated by the UKPDS risk engine according to the patient's sex, age at diagnosis of diabetes, smoking, systolic blood pressure, hemoglobin, TC, HDL-c, duration of diabetes, atrial fibrillation, and race [12].

### 2.6. Statistical Analysis

The SPSS 20 statistical software was used for data analysis. Data were expressed as mean ± standard deviation or mean (95% confidence interval). Categorical variables were presented as frequencies, with percentages given in parentheses. The CHD and stroke risks were assessed after stratifying patients by PAD status and age. We used the Mann-Whitney test or independent *t*-test to compare continuous variables among groups and the chi-square test to compare proportional data. Categorical parameters and risk estimation were evaluated using the chi-square test. Binary logistic regression analysis was used to analyze correlations between categorical variables and risk factors, and multivariate linear regression analysis was used for continuous variables. All statistical tests were two-tailed, and *P* < 0.05 was considered significant.

## 3. Results

### 3.1. Comparison of General Characteristics between Diabetic Patients with PAD and with non-PAD

Of the 1178 T2DM patients included in this study, 621 were men and 557 were women. Their average age was 58.1 ± 12.7 years (range, 21–90 years), and the mean duration of diabetes was 7.6 ± 6.6 years (range, 0–36 years). In total, 88 (7.5%) patients were assigned to the PAD group, and 1090 (92.5%) patients were included in the non-PAD group based on their ABI values. Among the 88 patients in the PAD group, 81 (6.9%) had an ABI ≤ 0.9 and 7 (0.6%) had an ABI > 1.4. Age; duration of diabetes; systolic blood pressure; hypertension rate; and the use of hypertension drugs, ACEI (angiotensin-converting enzyme inhibitor)/ARB (angiotensin receptor blocker), and statins were significantly higher in the PAD group than in the non-PAD group (*P* < 0.05 for all; Table 1). Sex distribution significantly differed between the two groups, with female patients being much more likely to have an abnormal ABI and therefore be included in the PAD group (*P* < 0.05).

### 3.2. Relationship of PAD with CHD and Stroke Risks

CHD risk, fatal CHD risk, stroke risk, and fatal stroke risk were significantly higher in the PAD group than in the non-PAD group (*P* < 0.05 for all; Table 2). Spearman correlation analysis indicated that the ABI was negatively correlated with age (*r* = −0.144, *P* < 0.01), CHD risk (*r* = −0.066, *P* < 0.01), stroke risk (*r* = −0.116, *P* < 0.01), and diabetes duration (*r* = −0.069, *P* < 0.05), while it was positively correlated with diastolic blood pressure (*r* = 0.078, *P* < 0.01) and BMI (*r* = 0.075, *P* < 0.05). The ABI was not correlated with HbA1c, systolic blood pressure, TC, TG, HDL, and LDL.

**Figure 1:**
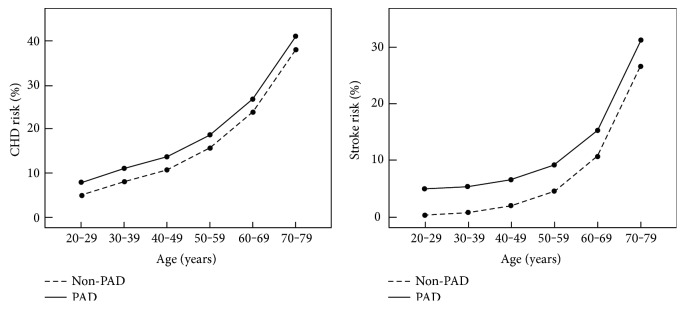
Age-related prevalence of CHD risk and stroke risk in diabetes patients. CHD: coronary heart disease; PAD: peripheral arterial disease; ABI: ankle-brachial index. PAD group: ABI ≤ 0.9 or ABI > 1.4; non-PAD group: 0.9 < ABI ≤ 1.4.

Considering that age is the most important factor affecting the ABI and CHD and stroke risks [19], we stratified the patients by age, in groups of 10 years, and calculated the UKPDS risk scores in both study groups (Figure 1). The results revealed that CHD and stroke risks gradually increased with age in both the PAD and non-PAD groups. Furthermore, the CHD risk, fatal CHD risk, stroke risk, and fatal stroke risk were higher in the PAD group than in the non-PAD for each age group.

### 3.3. Effect of PAD on the UKPDS Risk

The UKPDS CHD risk, fatal CHD risk, stroke risk, and fatal stroke risk were used as the dependent variables, and age, diabetes duration, PAD, HbA1c, TC, TG, HDL, LDL, BMI, systolic blood pressure, diastolic blood pressure, smoking, and sex were used as independent variables in a linear regression analysis. The results showed that age, diabetes duration, PAD, and sex were included in the linear regression equation (Table 3). We then performed a binary logistic regression analysis with the following dependent variables: age > 50 years, PAD, elevated HbA1c (≥ the average value 9.61%), hypertension, smoking, reduced blood HDL-c levels (<1.04 mmol/L (men) or <1.29 mmol/L (women)). The independent variables were as follows: UKPDS CHD risk (>20%, high risk, 1; ≤20%, 0) and stroke risk (>10%, high risk, 1; ≤10%, 0). The results showed that PAD was an independent risk factor for CHD (odds ratio: 3.6, 95% CI: 2.2–6.0, *P* = 0.000) and stroke (odds ratio: 6.9, 95% CI: 4.0–11.8, *P* = 0.000; Table 4).

## 4. Discussion

The ABI is a simple, inexpensive, and noninvasive method of detecting lower extremity PAD in diabetes patients. Various ABI cutoffs have been proposed for detecting PAD in different studies. The 2011 ACCF/AHA guidelines set the ABI cutoff at ≤0.9; in addition, they stated that an ABI > 1.3 suggested atherosclerosis, while an ABI > 1.4 indicated cardiovascular risk [3, 20, 21]. In this study, 81 (6.9%) patients had an ABI ≤ 0.9 and 7 (0.6%) patients had an ABI > 1.4; the rate of lower extremity PAD is lower than the rates reported previously [19]. This result could be due to inclusion of young diabetic patients and exclusion of the population with CHD and cerebrovascular disease in our study. The value of the ABI is related to age. According to Fowkes et al., in developing countries, the incidence of a low ABI among 45–49 year olds was 6.31% in women and 2.89% in men; in contrast, the incidence among 85–89 year olds was 15.22% in women and 14.94% in men [19].

This study showed that the proportion of women with a low ABI was significantly higher than that of men with a low ABI, which is consistent with previous literature [22, 23]. The San Luis Valley Diabetes Study showed that in the absence of traditional risk factors for cerebrovascular disease, the average ABI was 0.07 points lower in women than in men [22]. In the Multi-Ethnic Study of Atherosclerosis (MESA), which included patients without PAD and traditional atherosclerosis risk factors, the ABI was 0.02 points lower in women than in men after adjustment for multiple variables [23].

Our study also demonstrated that the 10-year CHD and stroke risks were significantly greater in the PAD group than in the non-PAD group, and the ABI was an independent predictor of the 10-year CHD and stroke risks. In addition to the diagnosis of PAD, the ABI is associated with cardiovascular risk factors and cardiovascular events. A low ABI has been related to many known cardiovascular risk factors, including hypertension, diabetes, smoking, dyslipidemia, obesity, and C-reactive protein [5–7]. Several population-based cohort studies have confirmed that a decrease in an ABI is highly correlated with the prevalence rate of coronary artery disease and cerebrovascular disease [8–11], which indicates that the ABI is an independent risk factor for cardiovascular and cerebrovascular disease. A few studies have indicated that an ABI > 1.40 is associated with stroke and CHD. The curve obtained by plotting ABI values on the *x*-axis and mortality and other cardiovascular events on the *y*-axis appears as a reverse J curve, in which the risk is lowest in the ABI range of 1.11–1.40 [3, 16, 24]. Reports have indicated that an abnormal ABI in patients with diabetes or metabolic syndrome is related to cardiovascular events and risk factors [1, 9, 16, 17]. A study from Hong Kong found that in diabetes patients, ABI values of 0.91–0.99 were associated with increased microvascular and macrovascular complications [18].

Considering that age is the most important factor affecting the ABI and CHD and stroke risks, we stratified the patients by age and recalculated the CHD and stroke risks. The results showed that the cardiovascular risk was higher in the PAD group than in the non-PAD group for every age group, which indicated that an abnormal ABI predicts CHD and stroke risks independent of age.

Furthermore, the combination of the ABI in cardiovascular risk stratification with the current methods for predicting the 10-year risk of cardiovascular disease would improve risk prediction. The ABI Collaboration conducted a meta-analysis of 16 cohort studies based on individuals, focusing on whether the ABI can predict the risk of cardiovascular events and death independently from the FRS and whether it can improve risk prediction when used in combination with the FRS [25]. The results showed that the use of the ABI would lead to a reclassification of the risk levels for men and women. This is consistent with the findings of the MESA study. The FRS is mainly used for the general population, while the UKPDS risk score is used for patients with diabetes [2]. There have been many studies on the correlation between the ABI and FRS in diabetes patients [26], but relatively few on the ABI and UKPDS risk score in diabetes patients. The ABI combining with the UKPDS risk engine for prediction of CHD and stroke risks in diabetes patients needs a further study.

This study has some limitations. First, the study involved only hospitalized patients, many of whom had poor glycemic control. However, at present, glycemic control is less than ideal all over the world. A cross-sectional study of 9065 T2DM outpatients from 26 medical centers in China found that blood glucose levels were controlled in only 32.6% of patients [27],which was similar to the rate of 31.78% among 238,639 diabetes patients reported by Ji et al. in 2013 [28]. The International Diabetes Mellitus Practice Study [29] included 11,799 patients from 17 countries in Eastern Europe, Africa, South America, and Latin America and found a blood glucose-control rate of only 25%. Thus, the glycemic control in our patients may reflect that observed in most diabetes patients. Second, the UKPDS risk engine originated from British diabetes patients, and whether or not it is suitable for Chinese patients remains to be investigated. However, it is currently an established risk assessment tool worldwide.

In conclusion, our study found that the 10-year CHD and stroke risks were higher in diabetes patients with lower extremity PAD than in diabetes patients without PAD, and lower extremity PAD was an independent risk factor for cardiovascular diseases in diabetes patients. Given that the ABI is a simple and easy method of detecting lower extremity PAD, ABI measurements will be beneficial for the estimation of cardiovascular disease and stroke risks in T2DM patients.

## Figures and Tables

**Table 1:** General characteristics of subjects.

	Non-PAD group	PAD group
Number	1090	88
Age (years)	57.2 ± 12.3	69.8 ± 11.8^#^
Gender (men/women)	586/504	35/53^∗^
YSDD (years)	7.3 ± 6.4	11.0 ± 7.8^#^
WC (cm)	87.9 ± 10.1	88.0 ± 10.2
BMI (kg/m^2^)	24.0 ± 3.6	23.3 ± 3.2
SBP (mmHg)	135.6 ± 19.5	145.4 ± 20.3^#^
DBP (mmHg)	81.8 ± 11.1	79.5 ± 12.8
HbA1c (%)	9.6 ± 2.4	9.5 ± 2.4
FBS (mmol/L)	9.2 ± 3.7	8.8 ± 4.1
Total cholesterol (mmol/L)	4.6 (4.5, 4.7)	4.5 (4.2, 4.8)
Triglycerides (mmol/L)	1.9 (1.9, 2.0)	1.9 (1.6, 2.2)
HDL-c (mmol/L)	1.2 (1.2, 1.3)	1.2 (1.1, 1.3)
LDL-c (mmol/L)	2.9 (2.9, 3.0)	2.9 (2.7, 3.2)
Hypertension (*n*, %)	(514, 47.2%)	(65, 73.9%)^#^
Smoker (*n*, %)	(368, 33.8%)	(26, 29.5%)
Nonantidiabetic drugs (*n*, %)	(184, 16.9%)	(6, 6.8%)^∗^
Only OAD (*n*, %)	(471, 43.2%)	(41, 46.6%)
Insulin + OAD (*n*, %)	(435, 39.9%)	(41, 46.6%)
Hypertension drugs (*n*, %)	(456, 41.9%)	(63, 71.6%)^#^
ARB/ACEI (*n*, %)	(227, 20.8%)	(38, 43.2%)^#^
Lipid-lowering drugs (*n*, %)	(157, 14.4%)	(18, 20.5%)
Statins (*n*, %)	(135, 12.4%)	(18, 20.5%)^∗^
Fibrates (*n*, %)	(20, 1.8%)	(0, 0%)

^∗^ *P* < 0.05 and ^#^*P* < 0.001 compared with the non-PAD group. Values are presented as the mean ± standard deviation; abnormal distribution values are shown as mean (95% CI).WC: waist circumference; YSDD: years since diagnosis of diabetes; BMI: body mass index (weight in kilograms/square of the height in meters); SBP: systolic blood pressure; DBP: diastolic blood pressure; FBS: fasting blood glucose; HDL-c: high-density lipoprotein cholesterol; LDL-c: low-density lipoprotein cholesterol; PAD: peripheral arterial disease; ABI: ankle-brachial index; OAD: oral antidiabetic drug; ARB: angiotensin receptor blocker; ACEI: angiotensin-converting enzyme inhibitors. PAD group: ABI ≤ 0.9 or ABI > 1.4; non-PAD group: 0.9 < ABI ≤ 1.4.

**Table 2:** Comparison of CHD and stroke risks.

	Non-PAD group	PAD group
*N*	1090	88
CHD risk (%)	20.5 (19.6–21.4)	35.1 (30.7–39.5)^#^
Fatal CHD risk (%)	15.1 (14.3–16.0)	29.7 (25.6–33.8)^#^
Stroke risk (%)	9.3 (8.6–10.0)	26.3 (21.7–30.9)^#^
Fatal stroke risk (%)	1.5 (1.3–1.6)	4.4 (3.5–5.4)^#^

^#^ *P* < 0.001 compared with the non-PAD group. Values are expressed as mean (95% CI). PAD was defined as an ABI ≤ 0.9 or >1.4. CHD: coronary heart disease; PAD: peripheral arterial disease; ABI: ankle-brachial index.

**Table 3:** Multivariate linear regression analysis of risk factors for CHD and stroke as estimated using the UKPDS risk engine.

Variables	UKPDS CVD risk		UKPDS stroke risk		UKPDS fatal CVD risk		UKPDS fatal stroke risk	
	Beta	*P*	Beta	*P*	Beta	*P*	Beta	*P*
Male	−0.355	0.000	−0.168	0.000	−0.304	0.000	−0.142	0.000
Age	0.726	0.000	0.602	0.000	0.711	0.000	0.507	0.000
BMI	−0.023	0.040	0.004	NS	−0.023	NS	−0.004	NS
Duration	0.081	0.000	0.270	0.000	0.148	0.000	0.248	0.000
SBP	0.082	0.000	0.044	NS	0.101	0.000	0.232	0.000
DBP	−0.020	NS	0.000	NS	−0.025	NS	−0.022	NS
HbA1c	0.352	0.000	0.031	NS	0.363	0.000	0.041	0.025
LDL−c	−0.024	NS	−0.022	NS	−0.016	NS	−0.022	NS
HDL−c	−0.285	0.000	−0.045	0.050	−0.233	0.000	−0.043	NS
TC	0.302	0.000	0.061	NS	0.237	0.000	0.050	NS
TG	0.000	NS	0.032	NS	−0.004	NS	0.024	NS
Smoking	0.051	0.000	0.019	NS	0.014	NS	0.018	NS
PAD	0.055	0.000	0.140	0.000	0.066	0.019	0.140	0.000

NS: not significant; BMI: body mass index; SBP: systolic blood pressure; DBP: diastolic blood pressure; HDL-c: high-density lipoprotein cholesterol; LDL-c: low-density lipoprotein cholesterol; PAD: peripheral arterial disease; ABI: ankle-brachial index; TG: triglyceride; TC: total cholesterol; HbA1c: glycosylated hemoglobin; UKPDS: United Kingdom Prospective Diabetes Study; CHD: coronary heart disease.

**Table 4:** Multivariate binary logistic regression analysis of risk factors for CHD and stroke as estimated using the UKPDS risk engine.

Variables	CHD risk		Stroke risk	
	OR (95% CL)	*P*	OR (95% CL)	*P*
Age ≥ 50 years	33.2 (20.2–54.4)	0.000	255.1 (35.5–1832.4.5)	0.000
Hypertension	2.0 (1.5–2.8)	0.000	2.1 (1.5–2.8)	0.000
Smoking	5.5 (3.9–7.6)	0.000	1.5 (1.1−2.0)	0.013
Elevated HbA1c	4.5 (3.4–6.1)	0.000	0.8 (0.6–1.1)	0.182
Reduced HDL-c	1.4 (1.1–1.9)	0.012	0.9 (0.7–1.2)	0.594
PAD	3.6 (2.2–6.0)	0.000	6.9 (4.0–11.8)	0.000

UKPDS: United Kingdom Prospective Diabetes Study; CHD: coronary heart disease. Elevated HbA1c (≥ the average value 9.61%); reduced HDL-c (<1.04 mmol/L (men) or <1.29 mmol/L (women)).
